# Evaluation of Computational Neoantigen Prioritization Strategies for Personalized Cancer Vaccines

**DOI:** 10.3390/vaccines14100857

**Published:** 2026-09-28

**Authors:** Vladimir Zyrin, Evgeniy Mozheiko, Ivan Valiev, Alexey Lazarev, Tigran Gevorkyan, Matvey Murashko, Konstantin Okonechnikov

**Affiliations:** 1 Bioinformatics and Big Data Analysis Laboratory, N.N. Blokhin National Medical Research Center for Oncology, Moscow 115478, Russiam.murashko@ronc.ru (M.M.); 2 Research Center for Artificial Intelligence in Healthcare, N.N. Blokhin National Medical Research Center for Oncology, Moscow 115522, Russia; t.gevorkian@ronc.ru

**Keywords:** cancer vaccines, neoantigen prediction, peptide prioritization, immunotherapy, vaccine target selection

## Abstract

**Background**: Identification of immunogenic neoantigens remains one of the major computational bottlenecks limiting the development of effective personalized cancer vaccines. Although various computational methods have been proposed for neoantigen discovery, there is little independent evidence regarding which approaches most effectively prioritize vaccine candidates using experimentally validated datasets. We focused on an independent multi-cohort benchmark of widely used neoantigen discovery pipelines and neoantigen scoring tools using publicly available datasets with experimentally validated immunogenic peptide–Major Histocompatibility complexes (MHC). **Methods**: In order to evaluate computational strategies used for selection of candidate vaccine peptides, five end-to-end neoantigen prediction pipelines and seven neoantigen scoring and prioritization tools were independently benchmarked using experimentally validated neoantigen datasets. End-to-end pipelines were evaluated on the TESLA cohort using raw WES and RNA-seq data from five patients, while individual prediction tools were additionally assessed on the TESLA, NCI, and HiTIDE validation cohorts comprising experimentally verified immunogenic and non-immunogenic peptide–MHC complexes. **Results**: NeoHunter achieved the highest overall recovery of experimentally validated neoantigens among the evaluated pipelines, while the scoring and prioritization tools NetMHCpan, MixMHCpred, and MHCFlurry generally showed the strongest ranking performance, although their relative performance varied substantially between datasets and patients. Precision–recall, average precision, and top K analyses demonstrated that no single predictor consistently dominated across cohorts and revealed a clear trade-off between candidate list size, precision, and recovery of validated neoantigens. Analysis of clinically relevant candidate selection thresholds demonstrated that restricting vaccine design to only the highest-ranked peptides substantially reduces sensitivity, whereas broader candidate selection improves recovery at the expense of increased experimental burden. **Conclusions**: These findings provide practical recommendations for computational selection of vaccine targets and highlight current limitations that must be addressed to improve the success of personalized neoantigen-based vaccines.

## 1. Introduction

Personalized cancer vaccines developed through the prediction of patient-specific neoantigens represent one of the most promising directions in modern cancer immunology. Neoantigens are peptides generated as a result of tumor-specific alterations, most commonly somatic mutations, and absent from normal tissues, which confers high specificity for eliciting an adaptive immune response [1,2,3,4]. The principal sources of neoantigens include single-nucleotide variants, insertions and deletions, chromosomal translocations and gene fusions, and alternative transcripts that alter protein amino acid sequences [5,6,7].

The formation of an immunogenic neoantigen is a complex multi-step biological process that extends well beyond the presence of a somatic alteration. The neoantigen source transcript must be expressed and translated, and the mutant protein must be processed by the antigen-processing machinery. In the case of major histocompatibility complex class I (MHC class I) presentation, intracellular proteins are degraded by the proteasome into peptide fragments, which are subsequently transported into the endoplasmic reticulum by the transporter associated with antigen processing (TAP), where they may undergo further trimming and bind to major histocompatibility complex class I (MHC-I) molecules. The resulting peptide–MHC class I (pMHC-I) complexes are then transported to the cell surface and can be recognized by T-cell receptors (TCRs) on CD8+ cytotoxic T lymphocytes. In parallel, tumor-derived proteins may also enter the MHC class II pathway through endocytic or autophagic processing, leading to presentation by peptide–MHC class II (pMHC-II) complexes and recognition by CD4+ T-cells [8,9]. In addition, professional antigen-presenting cells, particularly dendritic cells, can cross-present exogenous tumor antigens on MHC class I molecules, a process that is essential for priming effective antitumor T-cell responses [10]. Thus, computational neoantigen prediction constitutes an interdisciplinary problem requiring integration of genomic, transcriptomic, and immunological data [11].

Despite remarkable progress in personalized mRNA cancer vaccines, clinical efficacy remains highly dependent on the computational identification of truly immunogenic neoantigens. Because only a limited number of peptides can be incorporated into an individual vaccine formulation, accurate prioritization has become one of the most critical steps of vaccine design. Even though there is high availability of numerous individual tools and integrated computational pipelines for neoantigen prediction, their practical applicability and reproducibility remain subjects of active debate. Most published prediction methods for neoantigen scoring and prioritization demonstrate claimed effectiveness on internal training or partially overlapping datasets, whereas their behavior on independent test data, particularly under conditions of complete end-to-end analysis, often remains insufficiently characterized. Training data and prediction targets differ among computational methods. MHC-I binding and presentation models typically use binding affinity or eluted ligand data, whereas some immunogenicity models rely on Immune Epitope Database (IEDB) T-cell assay data, whose curation historically focused on infectious diseases and other non-cancer conditions. Consequently, tumor-derived neoantigens are comparatively underrepresented in such general purpose immunogenicity datasets. This domain imbalance raises uncertainty about their generalizability to cancer neoantigens and supports independent evaluation using experimentally validated cancer datasets [12]. To address these challenges, a wide array of computational approaches has been developed, ranging from machine learning-based algorithms for neoantigen scoring and prioritization, such as NetMHCpan, MHCFlurry, MixMHCpred, MHCnuggets, Neoapred, DeepImmuno, BigMHC, and NUCC [13,14,15,16,17,18,19,20], to integrated frameworks like pVACseq, which aggregate predictions from multiple tools to prioritize the most promising neoantigen candidates [21,22]. These programs typically function as components of larger bioinformatics pipelines designed for end-to-end neoantigen prediction from sequencing data, including workflows such as nextNEOpi, ScanNeo2, NeoDisc, RONC, and NeoHunter [23,24,25,26]. Importantly, despite the availability of several reviews summarizing existing neoantigen prediction methods [11,27,28,29], these analyses rely predominantly on reported performance metrics and do not include independent, head-to-head evaluation of the methods under consistent conditions.

To solve this issue, the present study provides a comparative analysis of the performance of the aforementioned tools for neoantigen scoring and prioritization and full pipelines in direct application. For benchmarking, we employed three published datasets that focused on whole-exome sequencing, RNA sequencing, and HLA typing for neoantigen prediction. Importantly, all these datasets included experimentally validated sets of immunogenic and non-immunogenic peptides [30,31,32]. Our study focuses on the reproducibility of sequencing data analysis for antigenic peptide identification, the accuracy of neoantigen scoring and prioritization, and the performance of computational immunogenicity prediction.

Evaluation of these tools using the validated cohorts enabled an objective assessment of their performance, comparison of obtained results with characteristics reported in the literature, and identification of methodological limitations that should be considered in future developments of the field. Here, we present the results obtained on benchmark datasets and discuss future perspectives for improving neoantigen prediction methodologies.

## 2. Materials and Methods

### 2.1. Data Sources

The pipeline comparison was performed using the publicly available dataset generated by the Tumor Neoantigen Selection Alliance (TESLA) study [30]. This dataset includes whole-exome sequencing (WES), RNA sequencing (RNA-seq) from three tumor samples derived from melanoma, and two non-small cell lung cancer patients. Access to one more case from the TESLA project was officially blocked due to patient data regulations, as was also noted in other studies.

The accuracy of neoantigen scoring and prioritization was estimated using TESLA and two additional datasets that provided a curated list of experimentally validated immunogenic and non-immunogenic peptide–MHC complexes, which were used as the reference set for evaluating prediction performance. The first additional dataset is a compilation of published datasets from the Surgery Branch of the National Cancer Institute (NCI) [31]. This cohort comprises data from 112 patients, primarily with skin cutaneous melanoma, colon and rectum adenocarcinoma, lung adenocarcinoma, and breast invasive carcinoma. The second additional dataset is the Human Integrated Tumor Immunology Discovery Engine (HiTIDE) cohort with sequencing and immunogenicity screening results for 11 patients with metastatic melanoma, lung, kidney, and stomach cancers who were enrolled in phase I clinical trials of adoptive cell therapy [32]. The HiTIDE manuscript was also the main source of the identified peptide materials from these two datasets.

All three benchmark datasets comprised HLA-I-restricted candidate peptides evaluated using HLA-I binding and/or T-cell reactivity assays. Accordingly, only MHC-I prediction outputs were analyzed, while MHC-II-restricted neoantigen prediction was outside the scope of this study. The NCI and HiTIDE validation cohorts were not identified as training datasets for the evaluated tools in our study. In contrast, TESLA-derived data contributed to the development or evaluation of all pipelines as well as to BigMHC and DeepImmuno neoantigen scoring and prioritization tools.

### 2.2. Neoantigen Prediction Pipelines

To evaluate the performance of end-to-end neoantigen discovery workflows, four publicly available pipelines were selected: nextNEOpi v1.5.1b, NeoHunter v1.0, NeoDisc 1.60, and ScanNeo2 v0.3.8. In addition, we included our newly developed pipeline RONC as described below. All these pipelines implement complete analytical workflows starting from sequencing data and producing ranked lists of candidate neoantigens. Each pipeline integrates several analytical stages, including somatic variant detection, transcriptomic filtering, HLA typing, peptide generation, neoantigen scoring, and prioritization.

All pipelines were executed using the default parameters provided in their respective software repositories or the recommended configurations described in the corresponding publications when such recommendations were available. No additional optimization of filtering thresholds or prediction parameters was performed beyond the recommended settings in order to preserve methodological consistency and reproducibility (Table S1).

### 2.3. RONC Neoantigen Prediction Pipeline

The software suite for identification and ranking of potentially immunogenic sequences, hereinafter referred to as the pipeline, is implemented on the Galaxy platform [33]. Its design follows the GATK Best Practices recommendations for data pre-processing and somatic short variant discovery [34]. The pipeline comprises three main stages: (i) data preparation and alignment of sequencing reads, (ii) somatic mutation calling, and (iii) ranking of the obtained peptide sequences.

During the first stage, normal exome and tumor exome reads are aligned to the reference genome (GRCh38) using the BWA aligner, while the tumor transcriptome is aligned with STAR [35,36]. The resulting alignments are recalibrated, and duplicates are removed.

In the second stage, somatic mutations are identified in the normal–tumor exome pair. Mutect2 and FilterMutectCalls, from the GATK suite, are employed for somatic variant discovery [37]. The set of validated mutations is then compared with RNA-seq data using the isovar tool. The output of this stage is a list of somatic mutations detected in the exome and expressed in the transcriptome.

The third stage involves ranking the selected sequences. Affinity of the mutant peptides to MHC class I molecules is predicted using an in-house algorithm, RoncMHCAnalyser. The method was developed based on a neural network architecture using experimentally characterized peptide–MHC interactions obtained from the IEDB. The developed model represents MHC molecules using 30 polymorphic amino acid residues within the peptide-binding domain and processes MHC and variable-length peptide representations using dedicated neural-network components. The set of MHC alleles is determined by OptiType [38]. The final ranking is established based on the expression level of the mutant alleles and their predicted MHC-I binding affinity.

### 2.4. Neoantigen Scoring and Prioritization

To evaluate individual neoantigen scoring and prioritization algorithms independently of the full pipelines, several widely used computational predictors were selected. These included NetMHCpan v4.1, MHCFlurry v2.0, MixMHCpred v3.0, MHCnuggets v2.4, BigMHC 1.0, DeepImmuno v1.0, and pVACseq v1.6.0, which represent different methodological approaches for neoantigen scoring and prioritization and, in some cases, peptide immunogenicity, as described in Table S2. The NUCC model was not included in the analysis because the source code required to run the predictor was not publicly available at the time of the study, and only the model description was accessible in the repository. RoncMHCAnalyser was excluded since it was designed as part of a pipeline and has additional input dependencies, such as gene expression, while Neoapred was excluded due to GPU requirements for usage. pVACseq was only tested on the TESLA datasets with available additional required input data computed.

All tools were executed using default settings or parameter values recommended in their original publications. Input peptide sequences and HLA allele information were derived from the combined set of candidate neoantigens as provided in corresponding published materials in order to maximize coverage of peptide–MHC pairs.

### 2.5. Ensemble Prediction

In addition to individual predictors, the pVACseq framework was used to generate ensemble predictions. This framework integrates several neoantigen scoring and prioritization methods and aggregates their results to prioritize candidate neoantigens based on consensus scoring and filtering criteria. pVACseq was executed using the default configuration recommended by the developers and produced a unified report containing ranking metrics and filtering results derived from the combined predictions. Furthermore, the prioritization process incorporated critical biological heuristics. Candidates were filtered by tumor mutant allele expression, variant allele frequency (VAF), and sequencing depth to ensure that top-ranked neoantigens were stably bound, highly expressed, and clonally prevalent.

### 2.6. Performance Evaluation

The performance of pipelines was evaluated using the experimentally validated neoantigen sets collected from three studies: the TESLA study with available raw read materials, where 535 epitopes were assessed for T-cell binding in patient-matched samples, and 34 of 535 tested peptide–MHCs were positive; the NCI study with 103 out of 19,087 epitopes positively validated; and the HiTIDE study that validated 41 out of 1552 as immunogenic. True positive rate was defined as the proportion of experimentally validated immunogenic peptides correctly identified by a given method across either all or top-selected predictions based on the affinity score values ranking. False positive predictions corresponded in the same manner to peptide–MHC pairs predicted as candidates but experimentally classified as non-immunogenic in the dataset.

For the comparison of neoantigen scoring and prioritization tools initially, performance metrics were calculated using the combined candidate peptide set generated by the evaluated pipelines in order to maximize the number of tested peptide–MHC pairs, while not-detected evaluated candidates were discarded due to the lack of required input information, e.g., genomic location of the antigen somatic variance.

To account for the strong class imbalance in the validation datasets, predictor performance was first evaluated using precision–recall (PR) analysis. Experimentally validated immunogenic peptides were considered positive cases, whereas experimentally tested non-immunogenic peptides were considered negative, while candidates without experimental validation were excluded. Precision was calculated as the proportion of true positives among all candidates predicted as positive, whereas recall was calculated as the proportion of experimentally validated positives recovered. PR curves were generated by progressively traversing candidates ranked according to each prediction score. Overall ranking performance was summarized using average precision (AP), calculated as the mean of precision values observed at ranks containing a positive candidate, thereby assigning greater weight to methods that place validated neoantigens near the top of the ranking. In addition, Precision@K and Recall@K were calculated independently for each patient at predefined candidate list sizes K. Precision@K represents the proportion of validated neoantigens among the top K ranked candidates, whereas Recall@K represents the proportion of all validated neoantigens for a given patient recovered within the top K. Patient-level values were subsequently summarized across each validation cohort.

As an additional inspection, the top 100, top 25, and top 400 affinity-scored candidates, including non-validated, were investigated per sample for each dataset as well as affinity scores across all candidates.

To systematically evaluate prediction performance across the diverse HLA alleles represented in the TESLA validation dataset, the pVACseq consensus results were partitioned by their respective HLA restrictions. For each individual HLA allele cohort, the prediction outcomes were cross-referenced with the experimental ground truth to calculate the relative proportions of validated (true positive rate, TPR) and non-validated (false positive rate, FPR) peptide–MHC interactions. A data matrix of these allele-specific performance metrics was constructed to analyze the overall distribution of prediction outcomes. To visualize these distributions, an annotated heatmap was generated.

## 3. Results

### 3.1. Study Design

A key element of the selected analytical strategy was the use of three benchmark cohorts, which contain experimentally validated immunogenic and non-immunogenic peptides. In contrast to most published comparisons that rely on synthetic or partially annotated datasets, such datasets provide a rare opportunity to evaluate the performance of computational pipelines and individual prediction tools using independent data with known experimental outcomes. This enables not only assessment of method sensitivity but also quantitative characterization of false-positive prediction rates, a factor of critical importance for practical and clinical applications.

Neoantigen discovery pipelines that are based on WES and RNA-seq data typically follow a multi-step workflow integrating genomic and transcriptomic analysis, while a central step of the pipeline is scoring the candidates based on the peptide–MHC binding affinity and recognition by T-cells, which serves as a key filter for prioritizing candidate neoantigens (Figure 1, left side). The neoantigens can be divided into several groups, depending on how they emerge and later are detected, including methods such as single-nucleotide substitutions, small or large indels, fusion genes, and transcriptome splicing alterations. Pipelines typically focus on various types of these antigens, providing full results (Table S1). The main input for the neoantigen scoring and prioritization tools is the formed neoantigen peptide sequences from these candidates (Table S2).

Based on these considerations, the comparison analysis was structured into two consecutive stages. In the first stage, “Part A”, we evaluated end-to-end computational pipelines capable of processing WES and RNA-seq data as input and automatically generating a final list of candidate neoantigens (Figure 1, right side). This analysis reflects a realistic clinical workflow, in which errors at any stage, including somatic variant calling, expression-based filtering, epitope selection, HLA typing, and neoantigen scoring and prioritization, directly affect the final results. In the second stage, “Part B”, an independent comparison of individual neoantigen scoring and prioritization tools was performed (Figure 1, right side) by also using ranking based on the estimated affinity for prioritization of top candidates as reflected in a real experiment. This strategy allowed separation of the contribution of neoantigen scoring and prioritization models from effects introduced by pipeline architecture and filtering strategies.

### 3.2. Performance of Neoantigen Prediction Pipelines

In contrast to specialized tools designed solely for neoantigen scoring and prioritization, full neoantigen prediction pipelines that operate directly from FASTQ sequencing data more closely reflect real-world workflows used in the development of personalized cancer vaccines. Such pipelines must account for the combined effects of multiple stages of data processing, including somatic variant detection, RNA-based filtering, epitope selection, HLA typing, application of multiple neoantigen scoring and prioritization tools, and final candidate ranking.

In this study, we performed an initial analysis of the TESLA dataset starting from sequencing reads and using five state-of-the-art pipelines: nextNEOpi, NeoDisc, NeoHunter, ScanNeo2, and RONC [23,24,25]. NeoHunter identified 30 of the 34 validated neoantigen candidates, whereas RONC detected 29 out of 34, NeoDisc and nextNEOpi detected 28 of 34, and ScanNeo2 identified 24 of 34 (Figure 2a). The overlap between pipelines in terms of correctly predicted neoantigens was up to 17 validated candidates shared among the pipelines, indicating substantial similarities in the identification of validated epitopes. Among the three validated neoantigens that were not detected by some pipelines, no obvious distinguishing features were observed. The comparison also revealed a relatively high number of false positive predictions across all evaluated pipelines: 284 of 501 for nextNEOpi, 252 out of 501 for RONC, 233 of 501 for ScanNeo2, and 219 of 501 for NeoHunter.

We further evaluated the ranking performance of the complete pipelines using precision–recall analysis and average precision (AP). The results revealed an important distinction between overall neoantigen recovery and prioritization efficiency. Recall and precision signals slightly varied between toolkits, while NeoHunter mostly represented the highest precision (Figure 2b). However, AP comparison (Figure 2c) demonstrated that even though ScanNeo2 recovered fewer validated candidates in total, it had the highest ranking performance, with a mean patient-level AP of 0.311. NeoHunter showed the second-highest pooled AP (0.274), followed by NeoDisc (0.256).

As a next step, top K analysis further demonstrated the ability of complete pipelines to prioritize experimentally validated neoantigens within restricted candidate lists (Figure 2d,e). NeoHunter had the highest precision up to top K = 40, while ScanNeo2 showed the strongest early-ranking performance, despite recovering fewer validated neoantigens overall than several other pipelines.

The computational time for the pipelines did not vary strongly and was approximately ~22 h per sample (Table S3), indicating potential application in medical use. Nevertheless, in some unique cases, pipelines were overusing computational resources, such as, for example, nextNEOpi, which exceeded the allocated memory limit RAM in two samples, probably due to application in adjustment for various antigen candidate sources, such as, for example, fusion calling.

Overall, the analysis indicates that all evaluated methods successfully identify a substantial proportion of experimentally validated immunogenic epitopes. However, a pronounced excess of false positive predictions was observed as well, exceeding 50% for nearly all tools.

### 3.3. Performance of Neoantigen Scoring and Prioritization from Pipelines Output

To evaluate the performance of peptide–MHC binding affinity prediction and neoantigen prioritization tools, we selected several widely used and recently published methods, including NetMHCpan, MHCFlurry, MixMHCpred, MHCnuggets, DeepImmuno, and BigMHC [13,14,15,18,19,20]. In addition, we included the pVACseq framework in the comparison, as it aggregates predictions from multiple methods and reports consensus results based on the intersection of individual predictors [21,22]. To distinguish the contribution of peptide prioritization algorithms from upstream analytical steps, we next evaluated individual neoantigen scoring and prioritization tools independently of complete prediction pipelines.

As the first step, the candidate peptide–MHC pairs generated by the evaluated pipelines from the TESLA cohort were pooled into a unified dataset to reduce dependence on the candidate selection strategy of any single pipeline and allow direct comparison of scoring algorithms using identical input candidates.

As in the pipeline comparison, initially the primary outcome measure was the proportion of validated and non-validated peptide–MHC pairs predicted by each method among the neoantigens experimentally tested in the TESLA study (Figure S1a). Among the evaluated tools, NetMHCpan, DeepImmuno, and MHCFlurry demonstrated the highest overall sensitivity while maintaining comparatively low false positive rates. In contrast, MHCnuggets exhibited substantially lower specificity. BigMHC achieved moderate coverage of validated candidates but was also associated with a low rate of false positive predictions. The consensus-based pVACseq approach achieved higher recovery of validated neoantigens than the individual prediction methods evaluated in this analysis.

Using the pVACseq predictions, we further compared the performance of neoantigen scoring and prioritization across different HLA alleles represented in the TESLA validation dataset (Figure S1b). The results indicate substantial variability in prediction accuracy across HLA alleles, although the distribution of allele representation strongly influenced the observed performance. In particular, the most prevalent allele in the dataset, HLA-A*02:01—accounting for approximately 47% of all tested epitopes—closely reflected the overall average ratio of validated and non-validated predicted candidates (Figure S1b).

### 3.4. Performance of Neoantigen Prioritization Under Different Candidate Selection Thresholds

Although affinity prediction models assign continuous scores to candidate neoantigens, practical personalized vaccine development requires selecting only a limited number of peptides for downstream vaccine design. We, therefore, evaluated predictor performance under clinically relevant ranking thresholds using three independent validation cohorts, including TESLA and two additional control cohorts with available experimental verification of target antigen peptides: the NCI dataset (*n* = 112) that contains 19,087 pMHC pair candidates, with 103 out of them experimentally confirmed to be immunogenic, and the HiTIDE dataset (*n* = 11) that contains 41 positive candidate antigen peptides out of 1552 tested in total.

To provide an appropriate evaluation of the highly imbalanced neoantigen prioritization task, we performed a precision–recall (PR) curve computation. The analysis revealed substantial dataset-dependent variability in predictor performance (Figure 3a). NetMHCpan demonstrated the highest PR-AUC in the TESLA cohort, whereas MixMHCpred and MHCFlurry showed the highest performance in the NCI and HiTIDE cohorts, respectively. BigMHC also demonstrated consistently strong performance across all three datasets. Overall, NetMHCpan, MixMHCpred, MHCFlurry, and BigMHC formed the best-performing group, whereas DeepImmuno and MHCnuggets generally showed lower precision–recall performance. Importantly, no single predictor consistently outperformed all other methods across all validation cohorts, highlighting the dataset-dependent nature of neoantigen prioritization performance.

We followed the inspection by checking the average precision across these tools (Figure 3b). This analysis confirmed substantial dataset-dependent variability in predictor performance. NetMHCpan achieved the highest pooled AP in TESLA (0.243), MixMHCpred in NCI (0.050), and MHCFlurry in HiTIDE (0.105), while BigMHC consistently ranked among the best-performing approaches across all three cohorts. Overall, NetMHCpan, MixMHCpred, MHCFlurry, and BigMHC formed a consistently higher-performing group, whereas DeepImmuno and MHCnuggets generally showed lower ranking performance. Considerable inter-patient variability was nevertheless observed for all predictors.

Top K analysis further demonstrated that predictor performance depended on the number of candidates retained (Figure 4). In the TESLA cohort, the leading predictors recovered approximately 69–85% of validated neoantigens within the top 25 candidates and more than 90% within the top 50. In the substantially larger and more imbalanced NCI cohort, BigMHC, NetMHCpan, MixMHCpred, and MHCFlurry recovered approximately 50–60% of validated candidates within the top 25 and 71–78% within the top 100, whereas DeepImmuno and MHCnuggets showed markedly lower recovery. HiTIDE demonstrated greater variability among predictors. Overall, increasing K improved recall while progressively decreasing precision, illustrating the trade-off between recovery of potentially immunogenic neoantigens and the number of candidates requiring downstream experimental validation.

For these datasets, we also followed the strategy of checking the binding affinity score computed by prediction tools for each peptide by taking into account non-validated peptides and then inspecting only the top 100 based on the affinity score values ranking to compute the proportion of validated and negatively identified among them. These intermediate vaccine formulation constraints were selected as an initial benchmark because this range is commonly used during experimental prioritization before downstream validation. Across all three validation datasets, prediction performance differed considerably between scoring algorithms, although several methods consistently ranked validated neoantigens near the top of their prediction lists. For the NCI dataset, we observed that three tools overtook a true positive rate close to 60% (Figure S2a): MixMHCpred, NetMHCpan, and BigMHC. The proportion of negative cases identified was only around 2% across all tools, with the only exception being DeepImmuno, where it went up to 4%. For the HiTIDE dataset, the results of comparison among tools demonstrated a similar pattern (Figure S2b): a top mean accuracy of close to 50% was achieved by MixMHCpred and NetMHCpan. Nevertheless, the number of false positives was observed to be much higher in this dataset—around 30% in general. The same inspection of the top 100 was performed for all identified peptides from the TESLA dataset as well (Figure S2c). There, MixMHCpred demonstrated the best result. The false positive rate was typically three times lower in comparison to the true positive rate for all tools except MHCnuggets, where it appeared to be slightly higher. Despite substantial differences in tumor type, cohort size, and validation strategy, NetMHCpan and MixMHCpred consistently ranked among the highest-performing predictors across all three datasets.

We also investigated the limit of only top 25 candidates selection, as strict vaccine formulation constraints more closely reflect the potential design of antigen-based vaccines in real hospital practice, and we observed a clear loss of true positive candidates, covering a maximum of 40% by MixMHCpred in the NCI dataset (Figure S3a), 22% by NetMHCpan and MHCFlurry in the HiTIDE dataset (Figure S3b), and 32% by BigMHC in the TESLA dataset (Figure S3c). Only adjusting to the top 400 allowed covering more than 80% of validated candidates in all datasets by NetMHCpan, MHCFlurry, and MixMHCpred, even though the false positive rate increased significantly as well (Figure S4). These results, in combination with recall–precision and top K inspection, demonstrate a clear trade-off between sensitivity and experimental burden: restricting candidate selection to smaller sets reduced the proportion of experimentally validated neoantigens recovered, whereas increasing progressively improved recall at the expense of precision.

Finally, we inspected the distribution of the affinity score rank of validated candidates across the full results without performing any top candidate selection (Figure S5). There, we also took into account ranking for peptides that were not investigated for validation. In general, the ranking pattern reflected observations from the comparison of the top 100, where NetMHCpan (Figure S5a), MixMHCpred (Figure S5b), and MHCFlurry (Figure S5c) clearly demonstrated lower mean ranking for validated cases in comparison to negative and not used in validation, while this pattern was not observed so clearly for other tools.

## 4. Discussion

Accurate identification of immunogenic neoantigens remains a central challenge in computational cancer immunology and is essential for the development of personalized cancer vaccines. Although numerous computational tools and pipelines have been developed for this purpose, their real-world performance is difficult to evaluate due to the limited availability of experimentally validated datasets. Only a few publicly available datasets provide a rare benchmark containing experimentally confirmed immunogenic and non-immunogenic peptide–MHC complexes, enabling independent assessment of prediction methods.

Based on this materials integration, our results show that modern neoantigen prediction pipelines can identify a substantial proportion of validated neoantigens. Among the evaluated workflows, NeoHunter demonstrated the highest sensitivity, followed by RONC, NeoDisc, nextNEOpi, and ScanNeo2. Conversely, from average precision and top K inspection, ScanNeo2 showed the strongest enrichment of validated neoantigens among highly ranked candidates, followed by NeoHunter and NeoDisc. These results demonstrate that overall neoantigen recovery and ranking efficiency represent distinct aspects of pipeline performance. In particular, pipelines recovering the largest total number of validated neoantigens did not necessarily concentrate these candidates most effectively at the top of their ranked lists.

Considerable inter-patient variability in AP was observed for all pipelines, indicating that relative performance also depends on individual tumor characteristics. Also, the overlap between the predicted sets of validated neoantigens was limited, indicating considerable methodological differences between pipelines. These discrepancies likely arise from variations in mutation calling, RNA-based filtering, epitope generation, and candidate prioritization.

An important limitation of the present study concerns the generalizability of the end-to-end pipeline comparison, which was performed using the TESLA cohort. Although this dataset provides one of the most extensively characterized publicly available datasets with experimentally evaluated neoantigens and matched sequencing data required for complete pipeline execution, the relatively small number of patients and validated immunogenic neoantigens limits the precision of performance estimates. Consequently, differences in recall and precision, AP, and top K recovery may be influenced by individual patients or peptides and should not be interpreted as establishing a universal performance ranking among the evaluated pipelines. Furthermore, the tumor-type and HLA composition of TESLA may not represent the broader diversity encountered in clinical populations, particularly for less frequent HLA alleles. The present pipeline-level results should, therefore, be interpreted as a comparative benchmark under the specific conditions represented by TESLA rather than as evidence that a particular workflow will consistently outperform others across cancer types and patient populations.

A consistent observation across all evaluated pipelines was the high number of false-positive predictions in the final output. While most tools were able to detect many validated neoantigens, the total number of predicted candidates greatly exceeded the number of experimentally confirmed epitopes. This behavior reflects a fundamental limitation of current prediction strategies. Most existing predictors are trained primarily on binding affinity or eluted ligand datasets, which capture antigen presentation but do not fully represent the biological mechanisms required for effective immune recognition.

A similar pattern was observed in the inspection of affinity prediction. Overall, the analysis indicates that all evaluated methods successfully identify a substantial proportion of experimentally validated immunogenic epitopes in top selected signals. However, a pronounced excess of false positive predictions ranging from 2% up to 40% was observed in the top 100 candidate selection. This behavior is consistent with known limitations of current prediction models. Binding and presentation predictors estimate peptide–HLA binding or the likelihood of presentation but do not directly model all downstream determinants of autologous T-cell recognition. In addition, some immunogenicity models are trained on heterogeneous T-cell assay datasets with limited representation of tumor neoantigens, which may constrain their generalizability to cancer-specific settings [12]. The high false positive rate may also reflect the limited number of negative examples in training datasets, as well as the underrepresentation of rare HLA alleles, features that are also characteristic of the patient cohort. In addition, model performance varies depending on peptide length (typically 8–11 amino acids), which may represent an additional source of variability. It should also be noted that the present top K analysis considers all experimentally validated neoantigens as equally relevant positive candidates. However, validated neoantigens may differ substantially in the magnitude and reproducibility of the induced T-cell response, tumor expression and clonality, antigen presentation, and other properties relevant to vaccine design.

A key limitation of current neoantigen prediction benchmarking is the strong reliance on a small number of datasets as the primary source of experimentally validated ground truth. For example, while TESLA represents one of the few publicly available datasets with matched sequencing data and systematic validation of peptide–MHC immunogenicity, its scope remains inherently limited. In particular, TESLA predominantly focuses on neoantigens derived from canonical somatic mutations (SNVs and indels) and includes a relatively small number of validated epitopes across a restricted set of tumor types. As a result, many existing prediction tools are effectively developed, tuned, or indirectly validated against a narrow representation of the neoantigen landscape, which may not generalize to broader biological contexts. Importantly, accumulating evidence indicates that a substantial fraction of clinically relevant neoantigens may arise from non-canonical sources that are not captured in TESLA. For example, recent studies have demonstrated the immunogenic potential of neoantigens derived from alternative splicing events, RNA editing, and non-coding regions [7,39,40]. Moreover, neoantigen prediction and validation have historically focused predominantly on MHC-I-restricted candidates, for which computational tools and validation frameworks are more established. Consistent with this emphasis, all three benchmark datasets examined here comprised HLA-I-restricted candidate peptides evaluated using HLA-I binding and/or T-cell reactivity assays. Consequently, this study did not assess MHC-II-restricted neoantigens or CD4+ T-cell responses, despite their importance in personalized cancer vaccination, and our findings should not be extrapolated to MHC-II neoantigen prioritization. These findings underscore the need for the development of new benchmark datasets that incorporate diverse sources of neoantigens. Expanding the diversity and scale of validation datasets will be essential for improving the robustness, generalizability, and clinical applicability of next-generation neoantigen prediction models.

Another factor influencing prediction performance is the composition of the training datasets used to develop these models. Databases such as the Immune Epitope Database contain predominantly pathogen-derived epitopes and relatively few tumor-associated antigens. This imbalance may limit the applicability of many predictors in the context of cancer neoantigens, and a potential solution is the establishment and integration of cancer-specific databases, such as, for example, the Cancer Epitope Database and Analysis Resource [41]. In addition, performance can vary across HLA alleles, partly due to the uneven representation of different alleles in both training datasets and benchmark cohorts. This problem also could be solved through systematic expansion of training and benchmarking datasets that could include a more balanced representation of HLA alleles, particularly rare and underrepresented variants, through coordinated data sharing and large-scale immunopeptidomics efforts.

The improved performance observed for the ensemble framework, such as pVACseq, further supports the idea that combining multiple prediction models can mitigate biases associated with individual predictors. Another clear verification of this idea is the development of a consensus prioritization framework that integrated multiple existing prediction methods and demonstrated improved neoantigen selection, although no new standalone prediction software was introduced [32]. Nevertheless, the favorable performance of pVACseq or other similar methods should be interpreted in the context of its consensus-based design rather than as direct superiority of an individual prediction algorithm. By integrating evidence from multiple predictors, consensus approaches can prioritize candidates supported by complementary prediction models and reduce dependence on the behavior of a single algorithm. However, increasing the number of incorporated predictors does not necessarily improve prioritization, since redundant or poorly performing models may introduce additional variability or dilute informative signals. These observations suggest that the benefit of ensemble approaches depends not only on combining multiple predictions but also on the selection and integration of individually informative models. Overall, our results highlight both the utility and the current limitations of computational neoantigen prediction methods and emphasize the need for improved training datasets and models that incorporate additional biological factors relevant to tumor immunogenicity.

## 5. Conclusions

In summary, our findings have direct implications for personalized cancer vaccine development, while current vaccine platforms typically include only a limited number of neoantigens, making computational prioritization one of the major determinants of clinical success. The observed differences between prediction tools indicate that computational method selection alone may substantially influence the final vaccine composition. Consequently, independent benchmarking studies, such as the present work, are essential for improving reproducibility and standardization of neoantigen selection workflows.

## Figures and Tables

**Figure 1:**
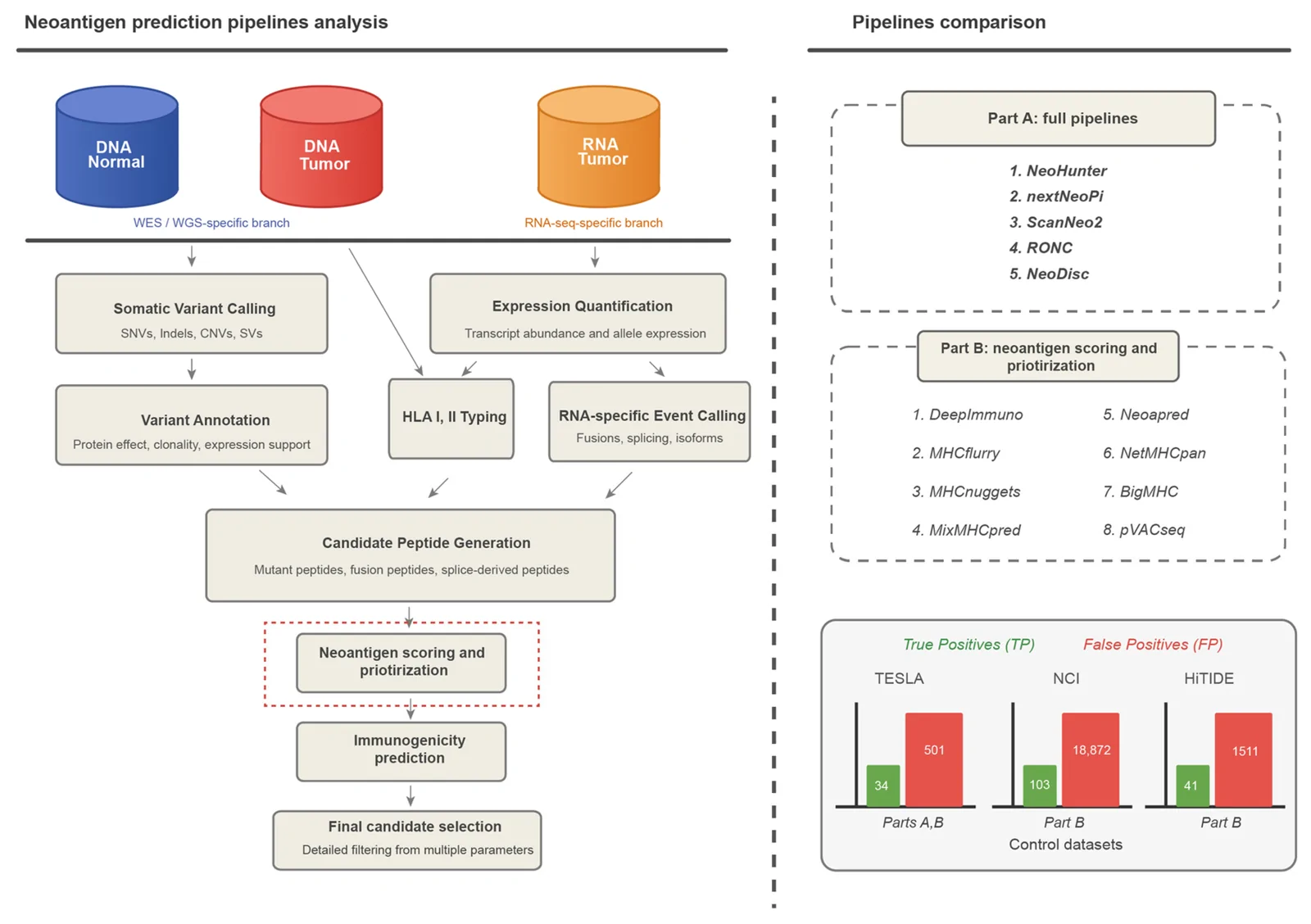
Study design and neoantigen prediction workflow. Schematic overview of the computational pipeline (**left side**) and benchmarking strategy (**right side**). Neoantigen prediction was performed on the TESLA dataset using tumor/normal DNA and tumor RNA sequencing data, with full pipeline usage steps including variant calling, HLA typing, neoantigen scoring and prioritization (highlighted in red dashed border line), and immunogenicity prediction. The arrows indicate data flow and processing sequence. End-to-end pipelines were evaluated on one dataset (TESLA), and individual neoantigen scoring and prioritization tools were evaluated on three datasets (TESLA, NCI, HiTIDE) using experimentally characterized peptide–MHC pairs and ranking-based performance measures, including precision–recall analysis, average precision, Precision@K, and Recall@K.

**Figure 2:**
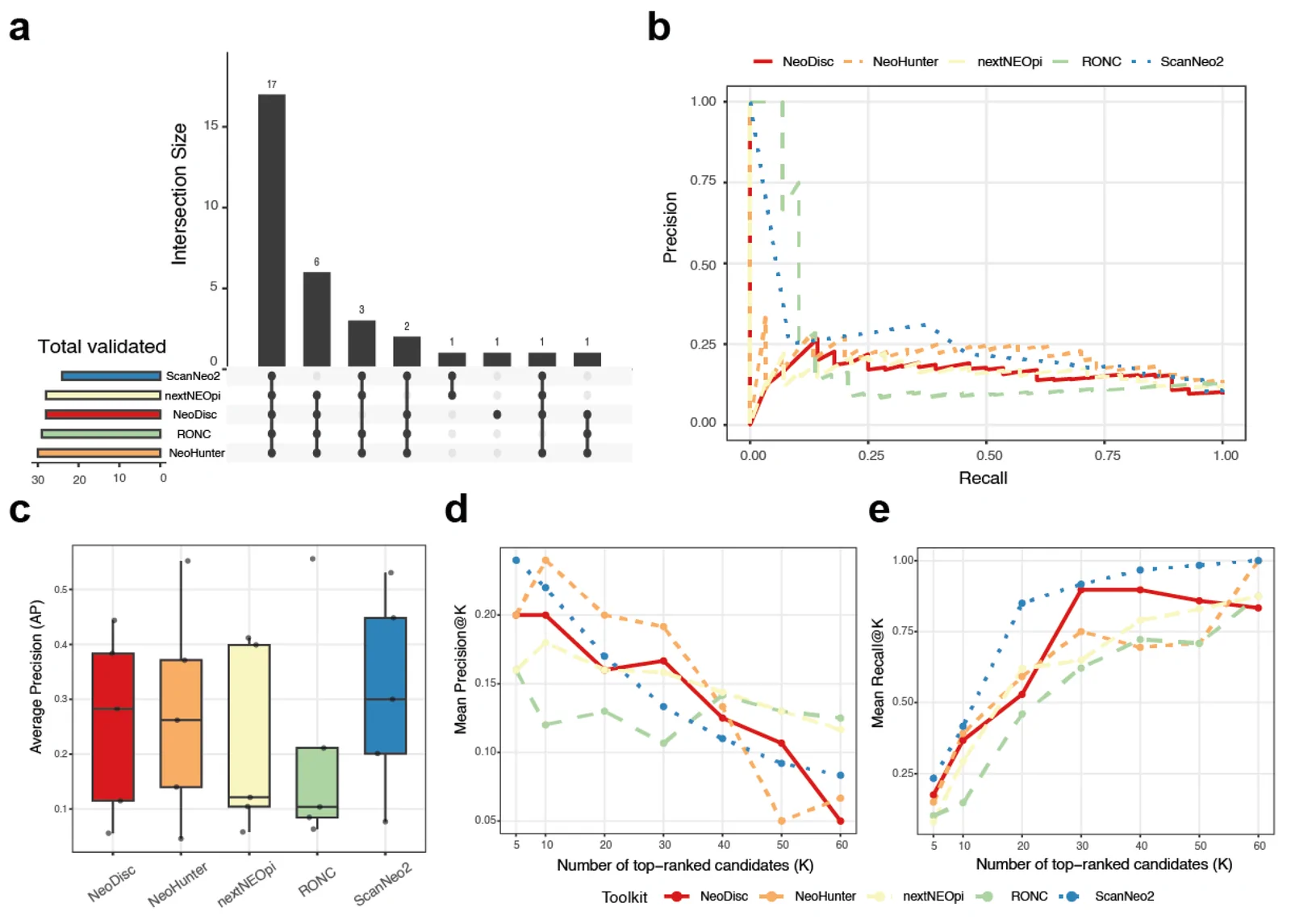
Performance comparison of neoantigen prediction pipelines on the TESLA benchmark dataset. (**a**) Upset plot demonstrates the distribution of validated peptides across the pipelines (NeoHunter, NeoDisc, RONC, nextNEOpi, and ScanNeo2). (**b**) Precision–recall curves. (**c**) Average precision boxplot. (**d**) Top k precision. (**e**) Top k recall.

**Figure 3:**
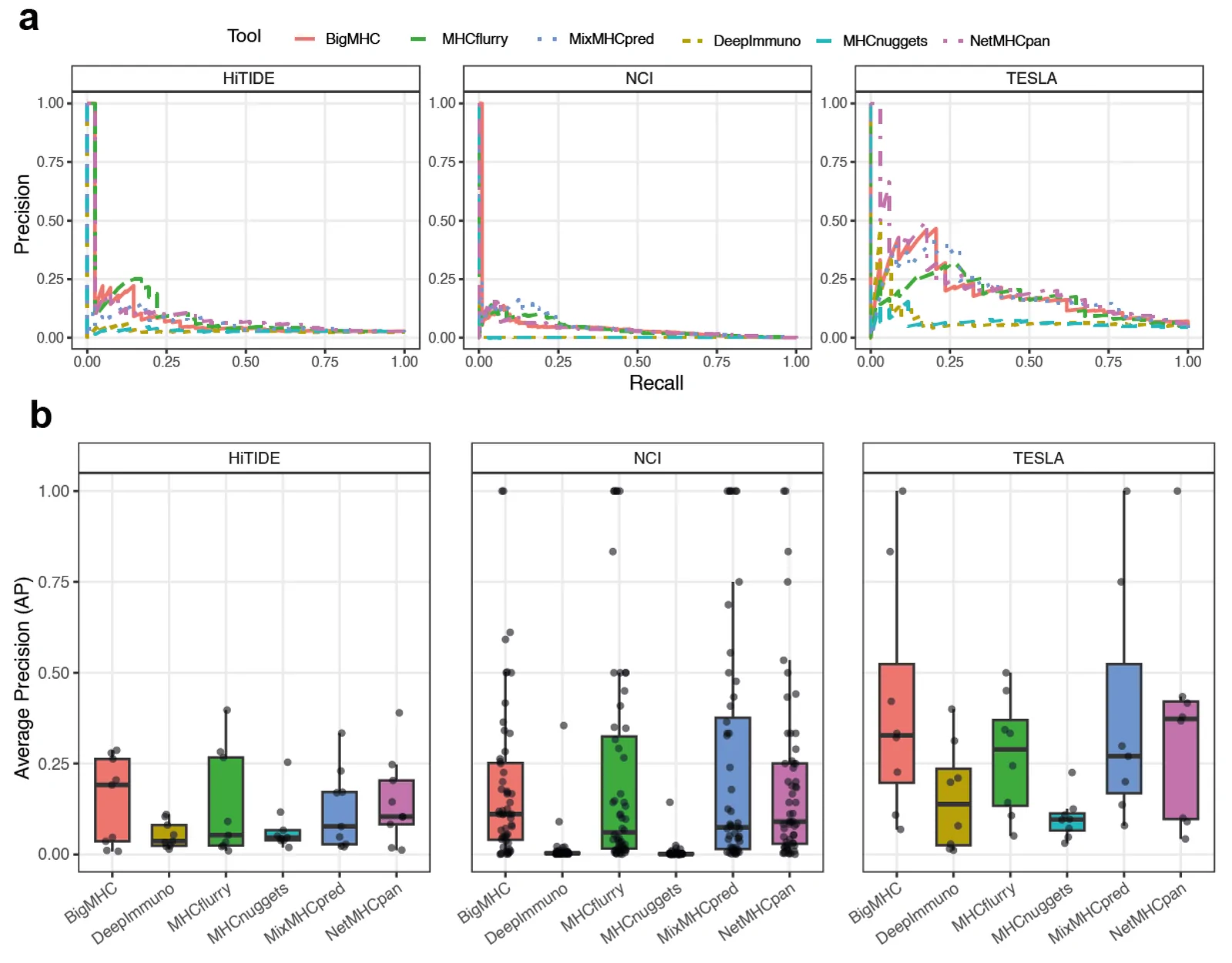
Statistical analysis plots for neoantigen scoring and prioritization tools on three control datasets: (**a**) precision–recall curves; (**b**) average precision boxplots. The target dataset name is in the title of the plot.

**Figure 4:**
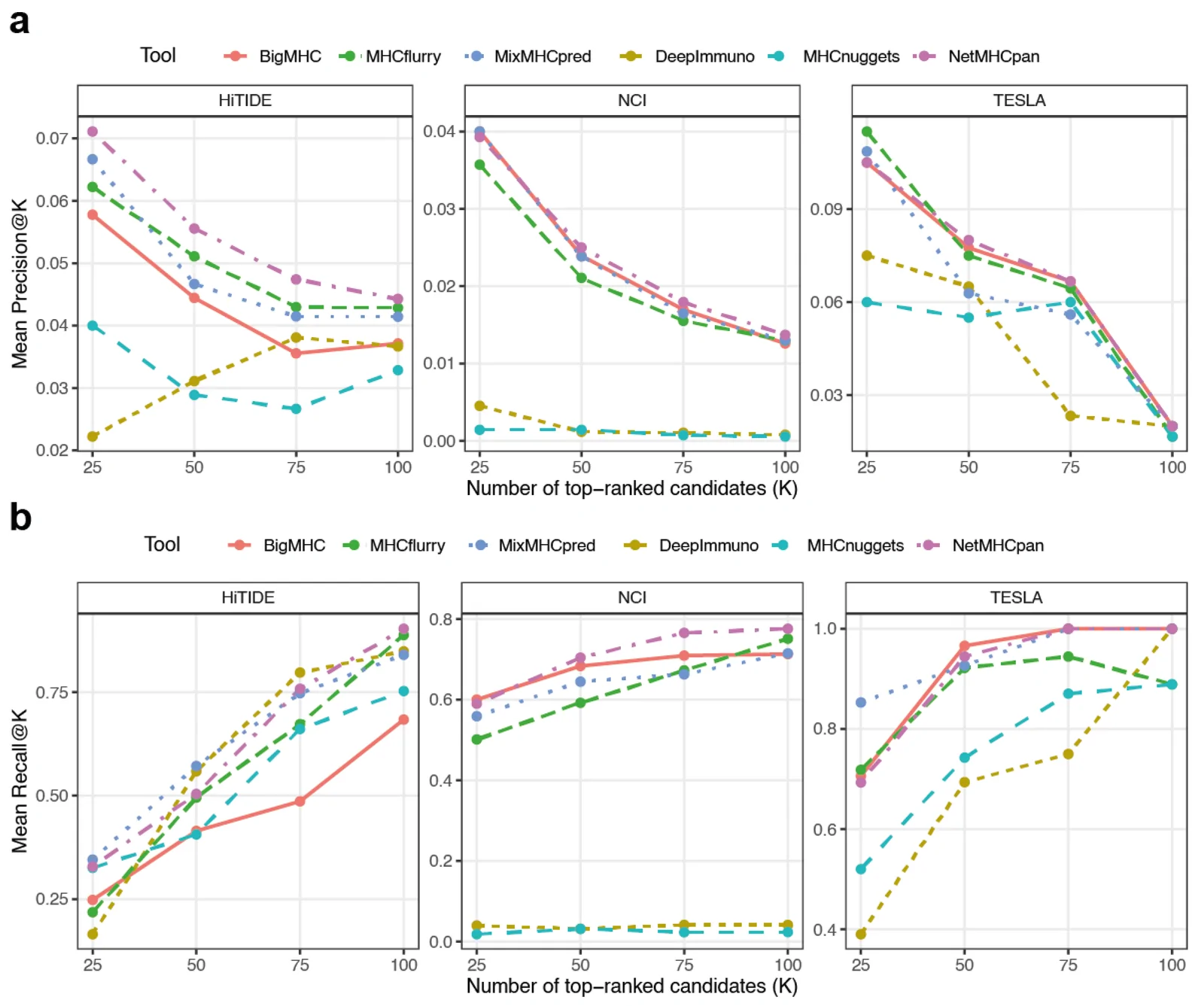
Statistical analysis plots for neoantigen scoring and prioritization tools on three control datasets: (**a**) Mean precision@K; (**b**) mean Recall@K. The target dataset name is in the title of the plot.

## Data Availability

The raw TESLA dataset analyzed for this study can be found in the Synapse repository (synapse.org, https://www.synapse.org/Synapse:syn21048999, accessed on 30 November 2025) under the ID syn21048999.

## Supplementary Materials

The following supporting information can be downloaded at: https://www.mdpi.com/article/10.3390/vaccines14100857/s1, Figure S1: (a) Comparison of prediction results for neoantigen scoring and prioritization tools for peptide selection from combined pipelines output. Bars represent the proportions of validated (TPR) and non-validated (FPR) peptide–MHC pairs among the candidates. (b) Distribution of prediction outcomes across different HLA alleles in the TESLA; Figure S2: Performance comparison of several neoantigen scoring and prioritization tools based on top 100 candidates selection from affinity score. Comparison of prediction results for neoantigen scoring and prioritization tools was performed on the (a) NCI, (b) HiTIDE, and (c) TESLA datasets. Bars represent the proportions of total validated (TPR) and non-validated (FPR) peptide–MHC pairs identified by the tools in the selection; Figure S3: Performance comparison of several neoantigen scoring and prioritization tools based on the top 25 candidates selection from affinity score. Comparison of prediction results for neoantigen scoring and prioritization tools was performed on the (a) NCI, (b) HiTIDE, and (c) TESLA datasets. Bars represent the proportions of total validated (TPR) and non-validated (FPR) peptide–MHC pairs identified by the tools in the selection; Figure S4: Performance comparison of several neoantigen scoring and prioritization tools based on the top 400 candidates selection from affinity score. Comparison of prediction results for neoantigen scoring and prioritization tools was performed on the (a) NCI, (b) HiTIDE, and (c) TESLA datasets. Bars represent the proportions of total validated (TPR) and non-validated (FPR) peptide–MHC pairs identified by the tools in the selection; Figure S5: Boxplots of neoantigen prioritization ranks computed via ordering all identified neoantigens by the tool-specific score for three merged datasets (NCI, hiTIDE, TESLA) with grouping for CD8-validated (named CD8), not validated, and other epitope pairs for (a) NetMHCpan, (b) MixMHCpred, (c) MHCFlurry, (d) MHCnuggets, (e) BigMHC, and (f) DeepImmuno tools; Table S1: Details about investigated pipelines including information about their analysis technique, training data source, implementation, and link to the repository; Table S2: Details about investigated tools including information about their analysis technique, training data source, implementation, and link to the repository; Table S3: Mean run time and resource usage of the neoantigen discovery pipelines launched on the TESLA dataset (*n* = 5) with the same server settings (*n* = 8 cores, 256 GB RAM per sample).
